# Physico-chemical characterization of African urban aerosols (Bamako in Mali and Dakar in Senegal) and their toxic effects in human bronchial epithelial cells: description of a worrying situation

**DOI:** 10.1186/1743-8977-10-10

**Published:** 2013-04-02

**Authors:** Stéphanie Val, Cathy Liousse, El Hadji Thierno Doumbia, Corinne Galy-Lacaux, Hélène Cachier, Nicolas Marchand, Anne Badel, Eric Gardrat, Alexandre Sylvestre, Armelle Baeza-Squiban

**Affiliations:** 1 Université Paris Diderot, Sorbonne Paris Cité, Laboratory of Molecular and Cellular Responses to Xenobiotics, Unit of Functional and Adaptive Biology (BFA) EAC CNRS 4413, Paris 75 013, France; 2 Laboratory of Aerology, University of Toulouse/CNRS, UMR5560, OMP, 14 Ave E, Belin, Toulouse 31400, France; 3 Laboratory of Climate and Environment Sciences (LSCE), CEA-CNRS, Orme des Merisiers, Gif sur Yvette cedex 91190, France; 4 Aix-Marseille University, CNRS, LCE FRE 3416, Marseille 13331, France; 5 Université Paris Diderot, Sorbonne Paris Cité, Therapeutic Molecules in silico, Inserm UMR-S 973, Paris 75 013, France

**Keywords:** 16HBE, Particle, Lung, Inflammation, Metabolism, Oxidative stress, Organic compounds, Traffic emissions, Domestic fires, Desert dust event

## Abstract

**Background:**

The involvement of particulate matter (PM) in cardiorespiratory diseases is now established in developed countries whereas in developing areas such as Africa with a high level of specific pollution, PM pollution and its effects are poorly studied. Our objective was to characterize the biological reactivity of urban African aerosols on human bronchial epithelial cells in relation to PM physico-chemical properties to identify toxic sources.

**Methods:**

Size-speciated aerosol chemical composition was analyzed in Bamako (BK, Mali, 2 samples with one having desert dust event BK1) and Dakar (DK; Senegal) for Ultrafine UF, Fine F and Coarse C PM. PM reactivity was studied in human bronchial epithelial cells investigating six biomarkers (oxidative stress responsive genes and pro-inflammatory cytokines).

**Results:**

PM mass concentrations were mainly distributed in coarse mode (60%) and were impressive in BK1 due to the desert dust event. BK2 and DK samples showed a high content of total carbon characteristic of urban areas. The DK sample had huge PAH quantities in bulk aerosol compared with BK that had more water soluble organic carbon and metals. Whatever the site, UF and F PM triggered the mRNA expression of the different biomarkers whereas coarse PM had little or no effect. The GM-CSF biomarker was the most discriminating and showed the strongest pro-inflammatory effect of BK2 PM. The analysis of gene expression signature and of their correlation with main PM compounds revealed that PM-induced responses are mainly related to organic compounds. The toxicity of African aerosols is carried by the finest PM as with Parisian aerosols, but when considering PM mass concentrations, the African population is more highly exposed to toxic particulate pollution than French population. Regarding the prevailing sources in each site, aerosol biological impacts are higher for incomplete combustion sources resulting from two-wheel vehicles and domestic fires than from diesel vehicles (Dakar). Desert dust events seem to produce fewer biological impacts than anthropogenic sources.

**Discussion:**

Our study shows that combustion sources contribute to the high toxicity of F and UF PM of African urban aerosols, and underlines the importance of emission mitigation and the imperative need to evaluate and to regulate particulate pollution in Africa.

## Background

A number of epidemiological studies have now established associations between exposure to particulate pollution and increased morbidity and mortality for respiratory and cardiovascular diseases [1]. Toxicological investigations in animals and humans have shown that the major short term effect of particle exposure includes lung and systemic inflammation [2] that in chronic condition is suspected to contribute to the exacerbation of chronic inflammatory diseases such as asthma and chronic obstructive pulmonary disease (COPD), particularly among vulnerable populations [3]. The fine and ultrafine fractions of the aerosol are now recognized as the more prone to induce biological effects due to their ability to reach the distal lung together with specific compositions including transition metals and organic compounds [4-6]. Particle toxicity results from their ability to trigger intracellular production of reactive oxygen species (ROS) in epithelial cells and macrophages, the first cells encountered by particles in the respiratory tract. This oxidative stress activates signalling pathways leading to the release of pro-inflammatory mediators (interleukins IL-8, IL-6; granulocyte macrophage colony stimulating factor GM-CSF) [7].

The effects of particles on health have been studied extensively in developed countries leading to specific regulations. Only a few studies have been conducted in developing countries such as in Africa [8,9]. This is in spite of the very high levels of pollution (both for gases – NO_2_, SO_2 _and particles) observed in African cities being at same levels as in Asian megacities [10-13] and well above WHO (world health organization) international norms. Such unexpected pollution is due to the explosive development of African megacities with largely unregulated traffic emissions including intensive use of 2 stroke vehicles [14] and very old vehicles, widespread domestic fires using wood, charcoal or animal waste, and finally in some countries by industries without norms or regulations. Such problems are expected to increase further in the near future due to the prolonged absence of any regulations since urbanization rates are known to be among the highest in the world. Moreover, this source of anthropogenic pollution is enhanced by other sources (e.g. desert dust and biomass burning (savanna fires) gases and aerosols, the impacts of which have been already underlined in Western African cities [9,11]. Improved knowledge of aerosol compositions, size and related biological reactivities are urgently needed since these sources which are associated with the intense photochemistry prevailing in Africa, are expected to generate pollution specificities and impacts, quite different from those in developed countries. Such studies could contribute to the proposal of mitigation options through identifying sources of concern.

In this context, the POLCA (Pollution des Capitales Africaines = African Capital Pollution) program has been jointly developed between African and French universities and institutes. One aim was to characterize atmospheric particulate pollution and to determine the toxic potential of particles according to their sizes (coarse, fine and ultrafine particles). Two traffic sampling sites were selected in African megacities: Bamako (Mali) and Dakar (Senegal). The sites differ in terms of the vehicle fleet, fuel type, road quality, domestic fires, dust events and biomass burning impacts. Within POLCA, experiments took place during the years 2008–2009 with intensive experiments occurring during the dry season (January and December 2009 in Bamako and Dakar respectively), a period well recognized to display maximum conditions of pollution in such areas [11].

In this paper, we display for the first time (i) results of an exhaustive size-differentiated physico-chemical characterization of African aerosols in these two cities exhibiting various specific sources of pollution, (ii) and characterization of the toxicity of three size-segregated aerosols in order to correlate their toxicities to specific sources. Three specific situations representative of pollution aerosol occurring at the two sites were scrutinized: Bamako with (BK1) and without (BK2) a desert dust event and Dakar (DK).

In order to screen size speciated aerosol compositions, coarse, fine and ultrafine PM from the two African cities have been sampled with different stage impactors for analysis of their total mass, organic and black carbon content, ion contents and trace elements. In addition, organic compounds such as polyaromatic hydrocarbons (PAH), polar compounds, water soluble organic carbon (WSOC) and light absorbing organic carbon (humic-like substances) were measured in bulk aerosol samples. In parallel, an impactor was devoted to collect aerosols for biological aspects. From this sampling, the toxicity of coarse, fine and ultrafine PM has been studied *in vitro* in human bronchial epithelial cells as relevant target cells. PM biological reactivities were characterized measuring the expression of a panel of biomarkers. Cytochrome P450 1A1 (CYP1A1) and NADPH quinone oxydoreductase (NQO-1), two xenobiotic metabolizing enzymes (XME) were investigated as exposure biomarkers that are induced after the uptake and metabolism of PM organic components by cells. Heme oxygenase 1 (HO-1), an antioxidant enzyme, GM-CSF and IL-6, two pro-inflammatory cytokines and amphiregulin (AREG), a growth factor were used as effect biomarkers of PM exposure for the occurrence of oxidative stress and pro-inflammatory response respectively. African aerosol reactivities were discussed by comparison with urban traffic aerosols in Paris that we previously studied using the same methodology and in which ultrafine and fine PM were shown to be the most reactive fractions [15,16].

## Results and discussion

In cities of developed countries, the health effects of particulate pollution are related to the finest particles which are mainly generated by traffic. By contrast the size dependent effect of PM from developing megacities devoid of specific regulations is still unknown. The high level of particulate pollution and the presence of multiple sources, some of which are absent in occidental cities could induce a different pattern of toxicity. Our study is the first one attempting to provide an extensive physico-chemical characterization of size-segregated aerosol samples in two different African megacities in association with a comparison of their biological reactivity towards human bronchial epithelial cells.

African aerosols were sampled at crossroads near the traffic and in two cities exhibiting different emission sources and geographical characteristics. Both cities, like the rest of African capitals, have rapid population grown (5% per year), which is known to be an important factor influencing pollution levels.

### Physico-chemical characterization of the aerosols

In Bamako city, traffic was dominated by gasoline and oil fuel vehicles especially motorcycles and domestic burning using fuelwood, charcoal and animal wastes. Moreover, Bamako is often exposed to Saharan dusts and is also impacted by trash burning and aerosols from unpaved roads. Dakar exhibited different traffic sources, mostly due to bigger vehicles such as minibuses using bad quality diesel. Dakar is also impacted by combustion aerosols coming from biomass burning, especially in winter. Note that Bamako is located in a basin in which the dispersion of pollutants is limited, while Dakar is a coastal site influenced by marine air masses that favour the dispersion of pollutants. Bamako and Dakar were characterized by high bulk PM concentrations as shown in Table 1. A higher concentration was obtained for the BK1 sample (205.8 μg.m^-3^) regardless of the size fraction that was linked to desert dust event influence. BK2 concentrations (122.1 μg.m^-3^) were higher than DK concentrations (80.7 μg.m^-3^). All these samples were composed mainly of coarse particles which comprised about 60% of the aerosol, compared with 10% for UF and 30% for F. Note that the concentrations of UF and F from DK were similar to PM_2.5_ concentrations found by the study of Dieme et al. [8].

**Table 1:** Gravimetric and source characteristics of Bamako and Dakar samples

**Samples**		**Bamako 1 (BK1)**	**Bamako 2 (BK2)**	**Dakar (DK)**
**Concentration μg.m**^**-3 **^**(Mass%)**	UF	18.0 (8.7%)	14.6 (12%)	6.0 (7.4%)
	F	57.1 (27.8%)	43.8 (35.9%)	24.1 (30%)
	C	130.7 (63.5%)	63.7 (52.1%)	50.5 (62.6%)
	Bulk	205.8	122.1	80.7
**Main sources**		Traffic, biomass burning, dust		Traffic, biomass burning
		Desert dust event	No desert dust event	No desert dust event

**Figure 1:**
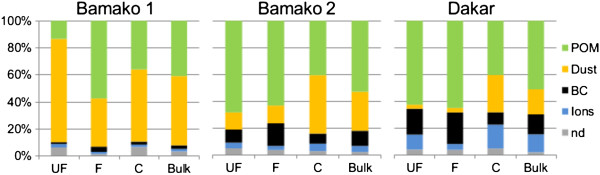
**Chemical mass closure for the 3 class fractions of PM for Bamako and Dakar samples.** For each site, the PM chemical composition of the 3 size fractions was totally determined by different analyses and results are presented as relative chemical abundance. Data are represented for Ultrafine PM (UF [0.03 μm-0.17 μm]), fine PM (F [0.17 μm-1 μm]), coarse PM (C [1 μm-10 μm]) and bulk aerosol. BC: Black Carbon; POM: Particulate Organic Matter; n.d.: not determined.

As expected in BK1, dust concentrations were higher in the coarse (76.8 μg.m^-3^) than in the UF (13.4 μg.m^-3^) and F (22.1 μg.m^-3^) modes. However, as shown in Figure 1, for the BK1 sample, the UF mode was largely dominated by mineral dust particles (76%), which was surprisingly much more important than in the coarse fraction, in which dust accounted for 53%. Particulate organic matter (POM) was the major component in the F mode, but dust still contributed significantly (36%). Black carbon (BC) and ions combined, represented 5% of the total aerosol mass. Note that the importance of dust was expected in BK1 due to a desert dust event occurrence during sampling. This scheme was very different for BK2 aerosol which was characterized by an important proportion of POM in both UF and F fractions (68 and 63%, respectively), while C mode mainly contained POM and dust (41 and 43%, respectively). As shown in Table 2, BC/OC ratio was relatively more important in BK2 than in BK1 in the F and C fractions.

**Table 2:** Average Black Carbon / Organic Carbon ratios (BC/OC) for Bamako and Dakar samples

	**BK1**	**BK2**	**DK**
**UF**	0.17	0.18	**0.49**
**F**	0.09	**0.35**	**0.54**
**C**	0.11	**0.34**	**0.36**
**Bulk**	0.10	0.31	**0.45**

The DK sample was dominated by carbonaceous aerosol (BC and POM), which accounted for 66% (Figure 1). The most abundant component was POM (51% in the total mass) but it was in the UF and F fractions that POM had the highest contribution (62% and 65%, respectively). BC relative content was higher in the UF/F modes than in Bamako, as shown by BC/OC ratio (Table 2). Note that this feature should be due to the relative importance of diesel vehicles in Dakar, while Bamako was dominated by more incomplete combustion sources. Such BC/OC ratio (0.45 in Dakar), was close to values of the order of 43%, given for urban European sites [17-20]. Sum of water soluble ions is more important in Dakar than in Bamako but less dust may be observed in Dakar that only represented 3% in UF/F modes. To summarize DK and BK2 samples showed a carbonaceous aerosol signature characterizing the combustion sources, while BK1 was more influenced by mineral dust probably from Sahel desert and unpaved roads as underlined by high amounts of Si, Al and Fe shown in the Table 3 concerning trace element concentrations. The averaged mass concentration of dust tracers (Al, Fe and Si) showed a decreasing gradient from BK1, BK2 and DK samples. However, the concentrations of Cu were highest in DK: this could be explained by industrial sources, which were not negligible because the site was located only 10 km from an industrial area.

**Table 3:** **Mean elemental concentrations (ng.m**^**-3**^**) for Bamako and Dakar samples**

**Sites**	**BK1**			**BK2**			**DK**		
Elements	UF	F	C	UF	F	C	UF	F	C
Ca	3.8	107.5	1935.7	17.7	116.7	964.2	8.8	72.3	1397.0
Na	8.9	69.9	735.0	9.1	35.9	155.6	48.4	104.9	1950.0
K	190.7	543.1	1627.9	178.8	391.2	381.0	95.8	151.2	264.7
Mg	1.8	80.9	873.6	5.8	42.7	233.5	1.4	19.1	390.2
**Al**	**388.6**	**553.1**	**6374.0**	**88.1**	**521.3**	**1865.4**	**267.3**	**76.9**	**858.0**
**Fe**	**17.1**	**272.3**	**3859.4**	**31.3**	**193.9**	**1750.8**	**11.4**	**59.4**	**806.7**
**Si**	**792.8**	**1128.3**	**13002.9**	**179.6**	**1063.4**	**3805.5**	**545.4**	**156.8**	**1750.3**
As	0.2	0.2	2.4	0.3	0.3	0.5	1.4	2.0	1.2
Ti	8.5	28.9	279.2	4.5	22.6	105.9	4.6	7.1	60.1
Zn	4.0	9.7	23.6	2.8	7.8	15.8	8.7	14.5	19.0
Ni	2.2	2.0	9.3	0.5	1.2	2.3	2.1	7.8	2.5
P	1.1	7.5	98.0	1.7	12.5	71.7	62.5	26.8	90.9
Pb	1.3	4.4	7.6	1.2	3.4	5.2	1.6	2.8	4.6
B	14.1	18.0	46.2	12.1	27.6	27.6	6.8	8.7	11.3
Mn	0.9	6.9	54.7	0.7	4.0	21.7	7.3	5.8	12.8
Sn	0.0	0.0	1.0	0.3	0.6	2.0	16.0	2.6	2.1
Cu	0.3	1.0	7.4	0.3	1.0	3.4	0.8	5.0	12.7
V	0.0	0.8	8.3	0.1	0.5	3.3	5.1	20.1	5.2

Major dust traces are in bold.

**Figure 2:**
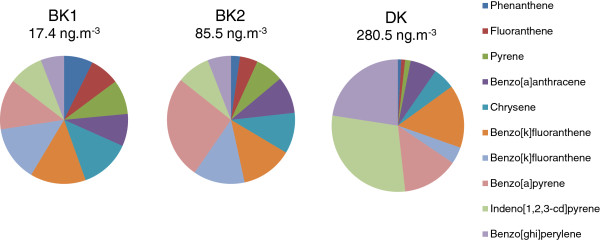
**Relative mass distribution of polyaromatic hydrocarbons (PAH) for bulk aerosol of Bamako and Dakar samples.** Results are expressed in percentage of the total PAH for each sample. The total quantity of PAH in the air is indicated in ng.m^-3^.

**Figure 3:**
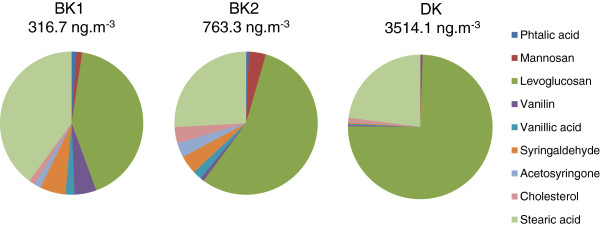
**Relative mass distribution of polar organic compounds for bulk aerosol of Bamako and Dakar samples.** The relative mass distribution of each sample was calculated for the bulk aerosol. The quantity of polar compounds in the air is indicated in ng.m^-3^.

Due to the relative importance of the organic fraction, other measurements were performed, with a more simple approach as already mentioned (bulk measurements only). They confirm the importance of source emissions on PM concentration:

– First, measurements of 10 PAH were obtained for BK1, BK2 and DK (Figure 2). Total amounts of PAH were quite different over the different sites with DK having the highest concentration (280.5 ng.m^-3^). At the BK site, the amount was different according to the sampling period and was particularly low for the BK1 sample (17.4 ng.m^-3^) characterized by a desert dust event and higher for BK2 (85.5 ng.m^-3^). Among these PAH, BaP African levels were much higher (factor of 7 for BK to 20 for DK) than those obtained in urban European sites such as Paris, Marseille and Grenoble (Ineris, 1998, N. Marchand, personal communication). In addition to these quantitative differences in mass, the relative distribution of the 10 PAH highly varied between Bamako and Dakar situations: DK was dominated by 3 PAH (IncdP: indeno[1,2,3-cd]pyrene, BghiP: benzo[ghi]perylene, BbF: benzo[b]fluoranthene) representing more than 2/3 of the total mass and BaP (benzo[a]pyrene) with 14%. 85% of total PAH were heavy PAH (5–6 cycles). This distribution profile and especially the relative abundance of BghiP were typical of diesel exhaust sources [21]. BK samples exhibited a more equally distributed pattern between the different PAH. Again, 4 PAH dominated the distribution (CHR: chrysene, BbF, BkF, BaP) each representing more than 12% and notably, BaP contributed to 29% of total PAH in BK2. The contribution of heavy PAH decreased to 61% for BK2 and 48% for BK1, compared to DK. Moreover, the relative importance of FLUA (fluoranthene), PHE (phenanthrene), PYR (pyrene) and CHR in BK samples, may be an indicator of the relative importance of incomplete combustion source e.g. biomass combustion [22,23] and oil and gasoline motorcycle [24].

– Second, the samples also differed in the relative abundances of organic polar compounds (Figure 3) with DK containing 3514.1 ng.m^-3^, 316.7 ng.m^-3^ for BK1, and 763.3 ng.m^-3^ for BK2. Levoglucosan was the predominant specie among those measured in the three samples. Stearic acid was more important in BK than DK due to domestic combustion (cooking) higher in BK than in DK. This assumption is also supported by a higher relative abundance of cholesterol in BK than in DK.

– Third, Water Soluble Organic Compounds (WSOC) presented higher values in BK than in Dakar as shown with WSOC/OC ratio higher in Bamako than in Dakar (Table 4) showing different solubility properties of components. This difference could be significant for the aerosol biological effect, as shown in Ramgolam et al. [15].

– Finally UV BC/IR BC (Ultraviolet BC/Infrared BC) ratios (Table 4) showed higher values in Bamako than in Dakar, which indicated a more important “light absorbing organic carbon” (refered as brown carbon) contribution in Bamako than in Dakar. Such a component could be partly formed by humic-like substances that was recently found to be the major redox active constituent of the water-extractable organic fraction in PM [25].

**Table 4:** Relative quantity of WSOC and UV BC/IR BC in Bamako and Dakar PM samples

	**Bamako**	**Dakar**
**WSOC/OC**	0.8	0.25
**UV BC/IR BC**	1	0.75

WSOC = water soluble organic carbon; UV BC/IR BC = light adsorption of black carbon (BC) in ultraviolets and infrared. Results are presented for bulk aerosols and globally for Bamako site.

### Site and size-dependence of the pro-inflammatory response

**Figure 4:**
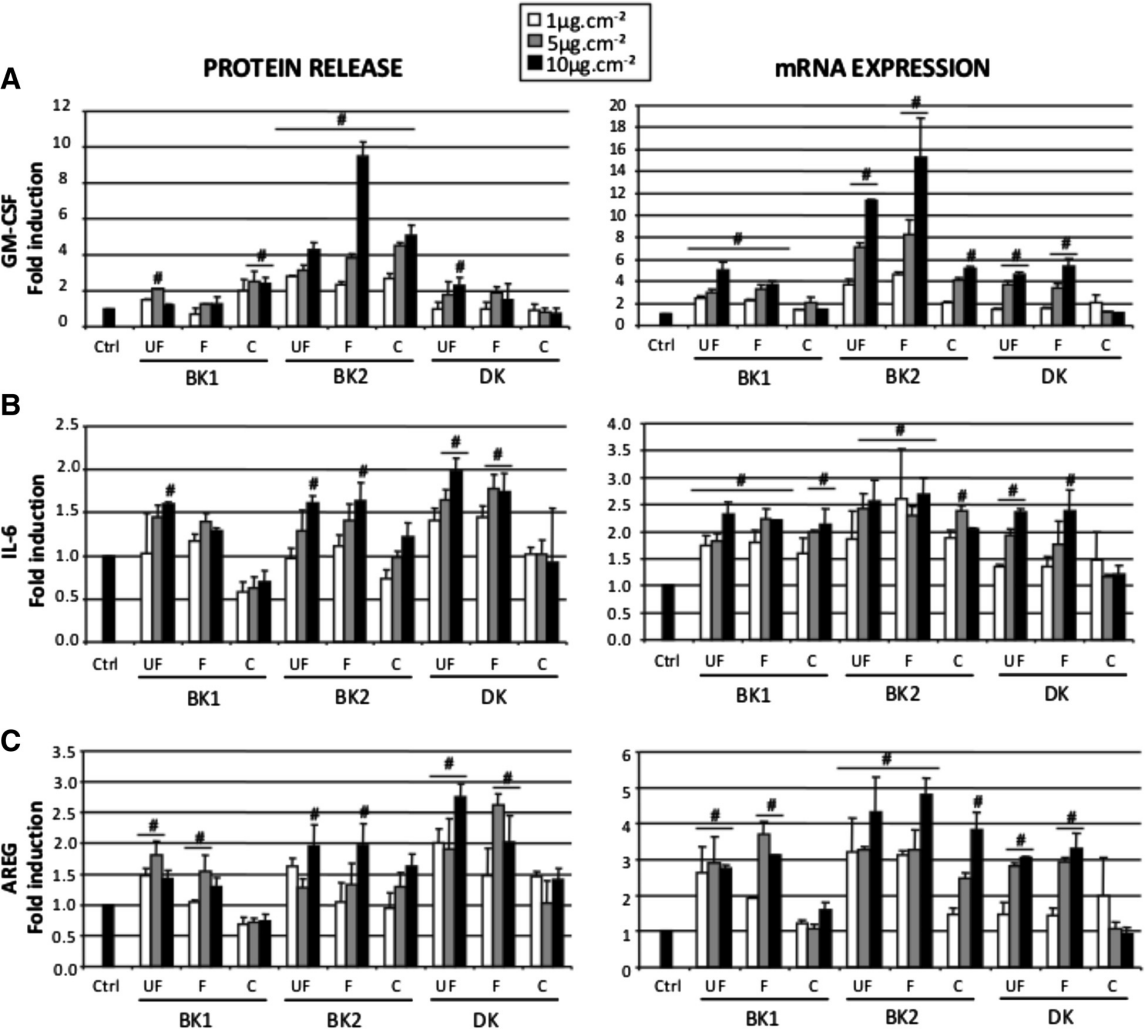
**Effect of PM on the expression and release of GM-CSF, IL-6 and AREG biomarkers.** Ultrafine PM (UF [0.03 μm-0.17 μm]), fine PM (F [0.17 μm-1 μm]) and coarse PM (C [1 μm-10 μm]) were used at 1 to 10 μg.cm^-2^ for 24 h exposures. The pro-inflammatory cytokines GM-CSF (**A**) and IL-6 (**B**) were studied as well as AREG growth factor (**C**). mRNA were evaluated by RT-qPCR and protein assessed in supernatants by ELISA assay. These data are expressed comparatively to the control (=1). mRNA and protein releases are mean of four replicates. * p < 0.05 compared with the control fixed to 1.

The pro-inflammatory effect of the size-segregated PM was investigated through the expression and release of two biomarkers (GM-CSF and IL-6) by 16HBE cells (Figure 4A, B). These pro-inflammatory cytokines have pleiotropic effects on the inflammatory process and act on inflammatory cell activation, recruitment, proliferation and survival [26,27]. They were also induced in asthma and chronic obstructive pulmonary diseases (COPD) [28-30], and after PM and nanoparticle exposure [16,31]. Whatever the site, UF and F PM dose-dependently induced mRNA expression of the two biomarkers with generally a significant effect from 5 μg.cm^-2^ and for BK samples from 1 μg.cm^-2^ (Figure 4A, B, right panel). The BK2 sample distinguished itself by the high GM-CSF fold inductions (more than 10) for UF and F at 10 μg.cm^-2^. By contrast, UF and F PM of BK1 and DK exhibited lower fold induction of GM-CSF mRNA. These data were consolidated with the measurement of the cytokine release especially for BK2 sample exhibiting again the highest GM-CSF release in a dose-dependent manner (Figure 4A, left panel). Nevertheless, C PM were not devoid of effects but their inductive potential towards GM-CSF expression mainly occurred with the BK2 sample. The presence of endotoxins frequently described as being associated to this size-fraction could explain the GM-CSF release. However due to the limited amount of PM dedicated to toxicological studies, it was not possible to run an endotoxin assay.

For IL-6 cytokine (Figure 4B), mRNA inductions (i) were less important than for GM-CSF (2.5 fold), (ii) were less clearly associated to a dose response effect according to samples (iii) were significant whatever size fractions excepting C PM from DK and (iv) were similar between BK2 and the other samples, making this biomarker less discriminating than GM-CSF. Considering PM-induced IL-6 release (Figure 4B, right panel), a better dose response was observed but fold inductions never exceed 2 and statistically significant effects only concerned the highest concentrations for F and UF PM.

The growth factor amphiregulin (AREG) was also studied as this ligand of the epidermal growth factor receptor plays a pivotal role in the repair and maintenance of epithelial tissues [32], but it is also overexpressed in asthmatic subjects during crisis [33]. Previous studies at the laboratory demonstrated that PM_2.5_ of different sites induced AREG mRNA expression in 16HBE cells and primary human bronchial epithelial cells NHBE [16,34] and was implicated in the persistence of GM-CSF pro-inflammatory response [35,36]. Consequently, its overexpression was suspected of participating in airway epithelium remodeling. AREG expression was induced whatever the samples with the UF and F fractions mostly from 5 μg.cm^-2^, C PM having a significant effect only for BK2 at 10 μg.cm^-2 ^(Figure 4C, left panel). As for GM-CSF, BK2 exhibited the highest fold induction (4 to 5) of AREG mRNA whatever the size fraction (Figure 4C, left panel) although it was not completely confirmed by the protein release (Figure 4C, right panel) as the stronger effect was observed with DK UF and F PM.

Considering the 3 biomarkers, GM-CSF is the one exhibiting the most discriminating effect among the different sites. This induction was previously associated with organic compounds of particles and their ability to induce reactive oxygen species (ROS) overproduction [36,37], an effect also evaluated in this study.

### Induction of biomarkers related to organic compounds metabolism and oxidative stress

**Figure 5:**
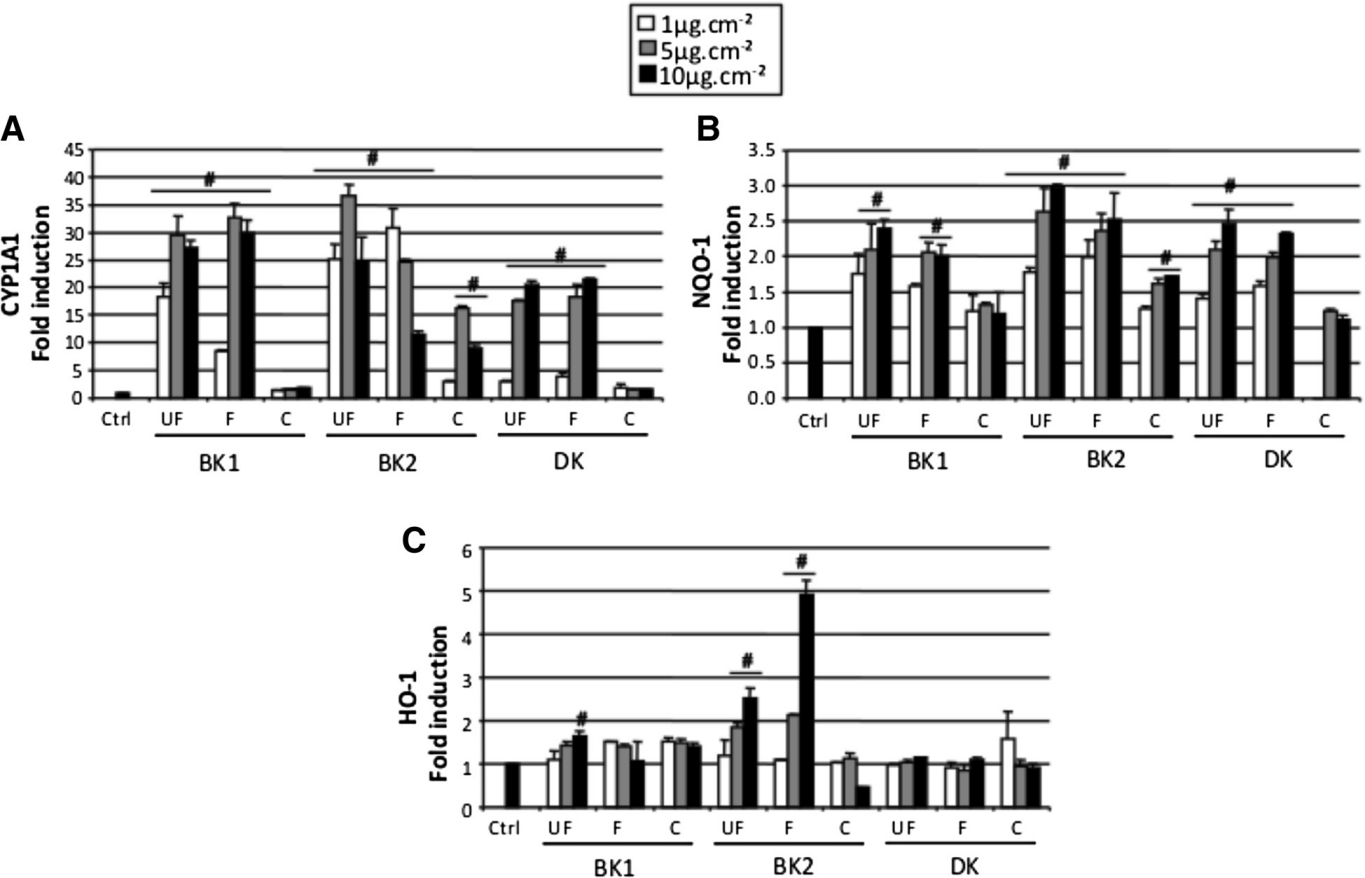
**Effect of PM on the expression of CYP1A1, NQO-1 and HO-1 biomarkers.** Ultrafine PM (UF [0.03 μm-0.17 μm]), fine PM (F [0.17 μm-1 μm]) and coarse PM (C [1 μm-10 μm]) were used at 1 to 10 μg/cm^2^ for 24 h. mRNA of CYP1A1 (**A**), NQO-1 (**B**) and HO-1 (**C**) were assessed. CYP1A1 and NQO-1 are metabolizing enzymes and HO-1 is an antioxidant enzyme. These data are expressed comparatively to the control (=1). Results are mean of four replicates. * p < 0.05 compared with the control fixed to 1.

CYP1A1 is a xenobiotic metabolism enzyme (XME) known to be specifically induced by PAH acting as an efficient ligand for Aryl Hydrocarbon Receptor (AhR) involved in transcriptional activation of CYP1A1 gene. As such it can be considered in our context as a biomarker of the PAH bioavailability [38]. Its activity produces electrophilic metabolites and reactive oxygen species contributing to the activation of NQO-1, another XME regulated by the antioxidant responsive element (ARE) [37] and HO-1, an antioxidant enzyme also containing ARE in its promoter [39]. Whatever the site, CYP1A1 was highly induced by UF and F PM (around 30 fold induction for 10 μg.cm^-2^ exposure) with significant effect from 1 μg.cm^-2^ and only to a lower extent by C PM only from BK2 (Figure 5A). CYP1A1 expression increased from 1 to 5 μg.cm^-2^ but did not further increase at 10 μg.cm^-2^. Surprisingly, F BK2 PM exhibited an inverse dose dependent effect.

NQO-1 showed similar profiles but with lower mRNA fold inductions (max 3) and was induced in a dose dependent manner (Figure 5B). Again among C PM, only those of BK2 had a significant effect.

The increase in the antioxidant enzyme HO-1 expression was only observed in few samples (Figure 5C). It was also for the UF and F PM of BK2 that the most striking effects were observed from 5 μg.cm^-2 ^(2.5 and 5 fold inductions for UF and F at 10 μg.cm^-2^ respectively). Otherwise UF PM of BK1 was the only other fraction inducing HO-1 at 10 μg.cm^-2^.

### Site dependent gene expression signature

**Figure 6:**
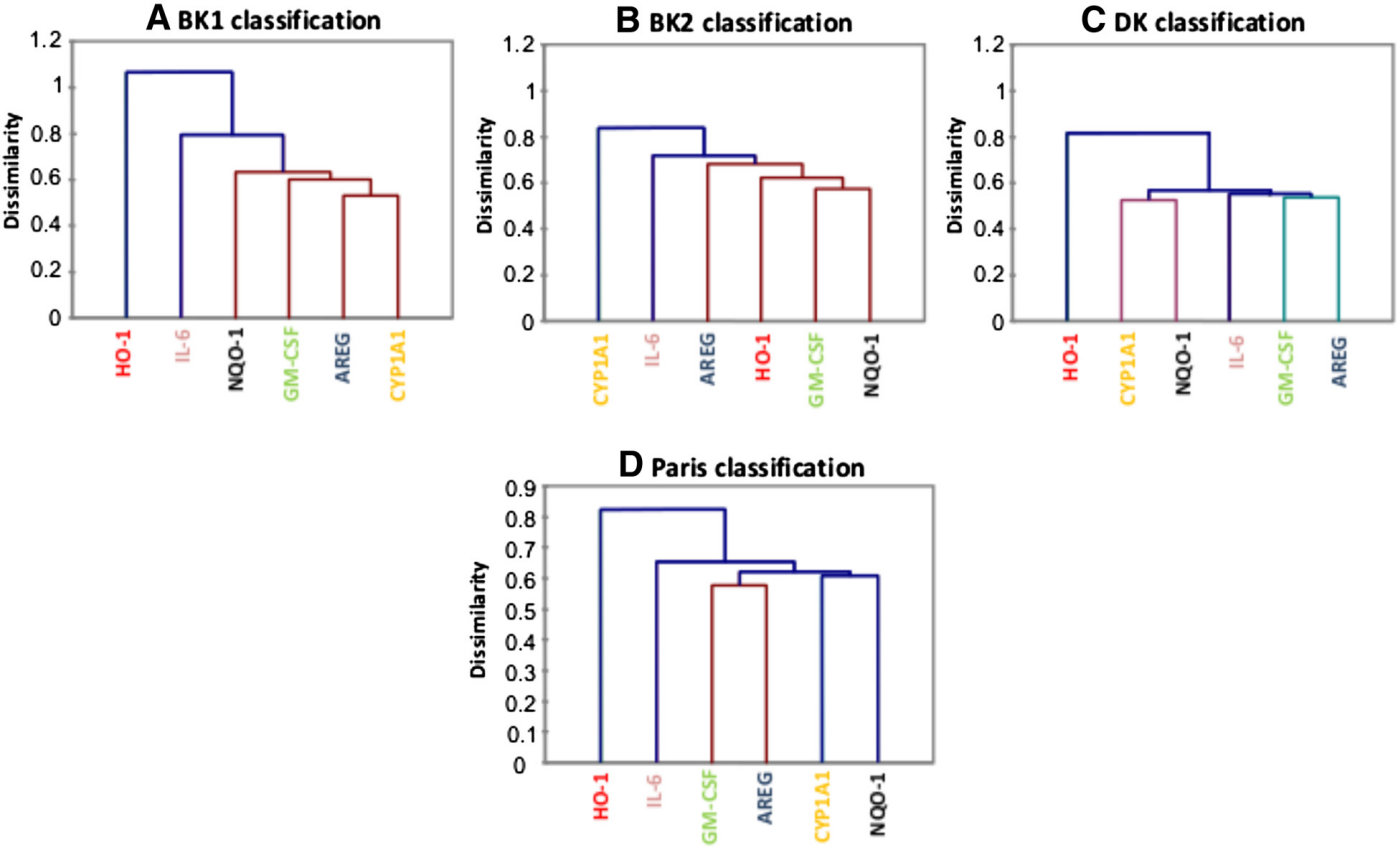
**Dendrogram classifications of biological responses for Bamako and Dakar samples in comparison with Paris samples.** Classifications were performed with Spearman’s dissimilarity with the mRNA results presented in Figure 4 and Figure 5 for BK1 (**A**), BK2 (**B**) and DK (**C**). Dendrogram classification for Parisian PM was performed with the results of Val et al. [16] (**D**).

In order to identify relationships between exposure biomarkers and effect biomarkers, correlation analyses were performed with the fold mRNA inductions shown in Figure 4 and Figure 5. For this purpose Pearson’s correlation factors (r) and dendrogram hierarchical classifications were performed (Table 5, Figure 6).

**Table 5:** Correlation coefficients (Pearson) between biomarker responses induced by PM exposure

**UF BK1**	GM-CSF	IL-6	AREG	CYP1A1	NQO-1	HO-1	**UF BK2**	GM-CSF	IL-6	AREG	CYP1A1	NQO-1	HO-1	**UF DK**	GM-CSF	IL-6	AREG	CYP1A1	NQO-1	HO-1
GM-CSF	**1**						GM-CSF	**1**						GM-CSF	**1**					
IL-6	**0.976**	**1**					IL-6	0.692	**1**					IL-6	**0.954**	**1**				
AREG	0.188	0.339	**1**				AREG	0.656	0.786	**1**				AREG	**0.965**	**0.940**	**1**			
CYP1A1	0.535	0.504	0.541	**1**			CYP1A1	−0.062	0.362	−0.024	**1**			CYP1A1	**0.986**	**0.943**	**0.983**	**1**		
NQO-1	0.599	0.422	−0.482	0.372	**1**		NQO-1	**0.899**	0.725	0.426	0.122	**1**		NQO-1	**0.972**	**0.961**	**0.940**	**0.951**	**1**	
HO-1	**0.880**	**0.872**	0.457	**0.838**	0.520	**1**	HO-1	**0.968**	**0.824**	0.791	0.073	**0.851**	**1**	HO-1	**0.885**	**0.852**	0.786	0.800	**0.914**	**1**
**F BK1**	GM-CSF	IL-6	AREG	CYP1A1	NQO-1	HO-1	**F BK2**	GM-CSF	IL-6	AREG	CYP1A1	NQO-1	HO-1	**F DK**	GM-CSF	IL-6	AREG	CYP1A1	NQO-1	HO-1
GM-CSF	**1**						GM-CSF	**1**						GM-CSF	**1**					
IL-6	**0.862**	**1**					IL-6	0.186	**1**					IL-6	**0.937**	**1**				
AREG	0.796	**0.870**	**1**				AREG	**0.888**	0.257	**1**				AREG	**0.847**	0.678	**1**			
CYP1A1	**0.896**	**0.882**	**0.968**	**1**			CYP1A1	**−0.922**	0.016	**−0.888**	**1**			CYP1A1	**0.913**	0.801	**0.963**	**1**		
NQO-1	0.680	0.740	**0.831**	**0.871**	**1**		NQO-1	**0.815**	0.361	0.533	−0.576	**1**		NQO-1	**0.974**	**0.891**	**0.925**	**0.951**	**1**	
HO-1	−0.723	−0.414	−0.308	−0.508	−0.279	**1**	HO-1	**0.963**	0.182	**0.931**	**−0.978**	0.676	**1**	HO-1	0.697	**0.849**	0.385	0.455	0.669	**1**
**BK1**	GM-CSF	IL-6	AREG	CYP1A1	NQO-1	HO-1	**BK2**	GM-CSF	IL-6	AREG	CYP1A1	NQO-1	HO-1	**DK**	GM-CSF	IL-6	AREG	CYP1A1	NQO-1	HO-1
GM-CSF	**1**						GM-CSF	**1**						GM-CSF	**1**					
IL-6	**0.611**	**1**					IL-6	**0.516**	**1**					IL-6	**0.948**	**1**				
AREG	**0.693**	**0.484**	**1**				AREG	**0.790**	**0.505**	**1**				AREG	**0.913**	**0.859**	**1**			
CYP1A1	**0.805**	0.442	**0.944**	**1**			CYP1A1	0.088	0.354	0.225	**1**			CYP1A1	**0.948**	**0.868**	**0.905**	**1**		
NQO-1	**0.812**	0.275	**0.675**	**0.843**	**1**		NQO-1	**0.799**	**0.566**	**0.650**	**0.543**	**1**		NQO-1	**0.717**	**0.665**	**0.610**	**0.827**	**1**	
HO-1	0.041	0.145	−0.224	−0.235	−0.092	**1**	HO-1	**0.911**	**0.477**	**0.640**	0.001	**0.620**	**1**	HO-1	0.200	0.322	0.322	−0.025	−0.393	**1**

Data are presented for UF, F and all size fractions (BK1, BK2, DK). Bold values represent a statistical correlation between biomarkers (p < 0.05).

BK1 and BK2 exhibited specific chemical closures leading to a lower biological reactivity of BK1 PM likely due to the dilution of the most toxic components by dusts. For BK1, cytokines/growth factor and CYP1A1 were highly correlated (Table 5, Figure 6A) underlying the importance of the combustion source in biological effects despite the high dust content. For BK2, closely related genes were GM-CSF and both HO-1 (0.97 for UF and 0.96 for F) and NQO-1 (0.9 for UF and 0.82 for F) which are two oxidative stress sensitive genes. Surprisingly GM-CSF induction was poorly correlated to CYP1A1 (0.08) due to a decrease in CYP1A1 induction with increasing PM concentrations (Table 5, Figure 6B) This result was confirmed in another human bronchial epithelial cell line, NCI-H292 (data not shown). Studies showed that several metals (copper, mercury and arsenic) were able to inhibit CYP1A1 mRNA induction in response to dioxin inductor TCDD [40-42]. However the highest concentrations of copper and arsenic were obtained in DK and not in BK2 (Table 3). Otherwise a specific metal speciation in BK2 sample and/or oxidative stress known to inhibit CYP1A1 [43] could explain the decrease of CYP1A1 mRNA for BK2 site at high PM concentrations which was related to HO-1 huge mRNA induction. Let us recall that BK presented more organic soluble fraction, and more “brown carbon” than DK, two organic indicators suspected for their oxidative properties [16,25,44]. The contribution of metals to oxidative stress is likely low. Indeed, the size fraction having the highest metal content was the coarse one (76 to 87% of the total metal mass) and yet was not or less reactive on 16HBE cells suggesting that the metallic component had a low toxicological implication.

For DK site, all the genes excepting HO-1 were closely linked (Figure 6C) suggesting a strong association between exposure biomarkers (CYP1A1 and NQO-1) and effect biomarkers (GM-CSF, IL-6 and AREG). It was driven by UF and F PM of DK (Table 5), as observed with PM sampled in Paris near the traffic [16] (Figure 6, Additional file 1). It could be explained by the predominance of diesel fuel cars in these two cities, even if there are old diesel engines in Dakar. These results target the diesel exhaust source in the biological response.

In summary, whatever the samples, carbonaceous aerosols from combustion sources seem to contribute to biological responses.

### Role of the organic component in the biological response

In order to specify which component in each size fraction of PM could be responsible to biological responses, correlations between genes fold inductions and major compound content were calculated (Table 6). The effects of UF and F PM seemed to be driven by BC and POM components for all the biomarkers, especially for GM-CSF and HO-1, but not for CYP1A1 as already discussed (Table 6A). But BC showed slight slope of lines in correlation graphs, suggesting its low implication.

**Table 6:** Correlation coefficients (Pearson) between biological responses and chemical analysis

**A**						
**UF**	GM-CSF	IL-6	AREG	CYP1A1	NQO-1	HO-1
BC	**0.960**	**0.677**	**0.686**	0.339	**0.789**	**0.849**
POM	**0.974**	**0.674**	**0.686**	0.411	**0.790**	**0.925**
Ions	0.084	0.299	0.099	−0.141	0.293	−0.177
Dust	−0.023	0.322	−0.013	0.157	0.303	−0.023
**F**	GM-CSF	IL-6	AREG	CYP1A1	NQO-1	HO-1
BC	**0.954**	**0.498**	**0.750**	−0.081	**0.717**	**0.940**
POM	**0.654**	0.444	**0.673**	0.238	**0.523**	**0.684**
Ions	−0.005	0.036	0.108	−0.028	0.296	−0.195
Dust	−0.058	0.095	0.205	0.210	0.311	−0.234
**Total**	GM-CSF	IL-6	AREG	CYP1A1	NQO-1	HO-1
BC	0.331	−0.076	0.050	−0.184	0.028	0.314
POM	**0.700**	**0.515**	**0.555**	0.258	**0.454**	**0.572**
Ions	0.092	0.106	0.166	0.058	0.277	−0.176
Dust	−0.022	0.181	0.089	0.123	0.237	−0.173
**B**						
**BK1**	GM-CSF	IL-6	AREG	CYP1A1	NQO-1	HO-1
BC	0.300	**0.607**	0.376	0.368	0.194	−0.359
POM	0.208	**0.607**	0.278	0.257	0.092	−0.329
Ions	**0.490**	**0.740**	0.245	0.311	0.317	0.012
Dust	**0.576**	**0.625**	0.173	0.287	0.413	0.251
**BK2**	GM-CSF	IL-6	AREG	CYP1A1	NQO-1	HO-1
BC	**0.913**	0.402	**0.759**	−0.167	**0.610**	**0.828**
POM	**0.863**	0.398	**0.768**	−0.024	**0.742**	**0.682**
Ions	0.348	0.105	**0.483**	−0.314	0.218	0.076
Dust	0.053	−0.057	0.256	−0.458	−0.129	−0.180
**DK**	GM-CSF	IL-6	AREG	CYP1A1	NQO-1	HO-1
BC	**0.869**	**0.793**	**0.712**	**0.853**	**0.762**	−0.075
POM	**0.781**	**0.718**	**0.601**	**0.770**	**0.714**	−0.099
Ions	−0.086	−0.063	−0.252	−0.073	0.052	−0.157
Dust	−0.433	−0.419	**−0.556**	−0.430	−0.286	−0.203

Bold values represent a statistical correlation between biomarkers (p < 0.05). Correlations were calculated for UF and F fractions whatever the site (A) and for each site whatever the size fraction (B). Total table was calculated with all results of Bamako and Dakar samples for the 3 class fractions.

Considering each site (Table 6B), BK1 (desert dust event) showed a correlation between cytokines and dusts but also IL-6 with BC and POM. By contrast BK2 and DK showed high correlations with BC and POM for 4 biomarkers.

Globally whatever the samples and size (Table 6, total), POM was the sole parameter correlating statistically with most of the biomarkers suggesting that biological effects were mainly driven by carbonaceous aerosols.

### Are African traffic aerosols more reactive than French ones?

**Figure 7:**
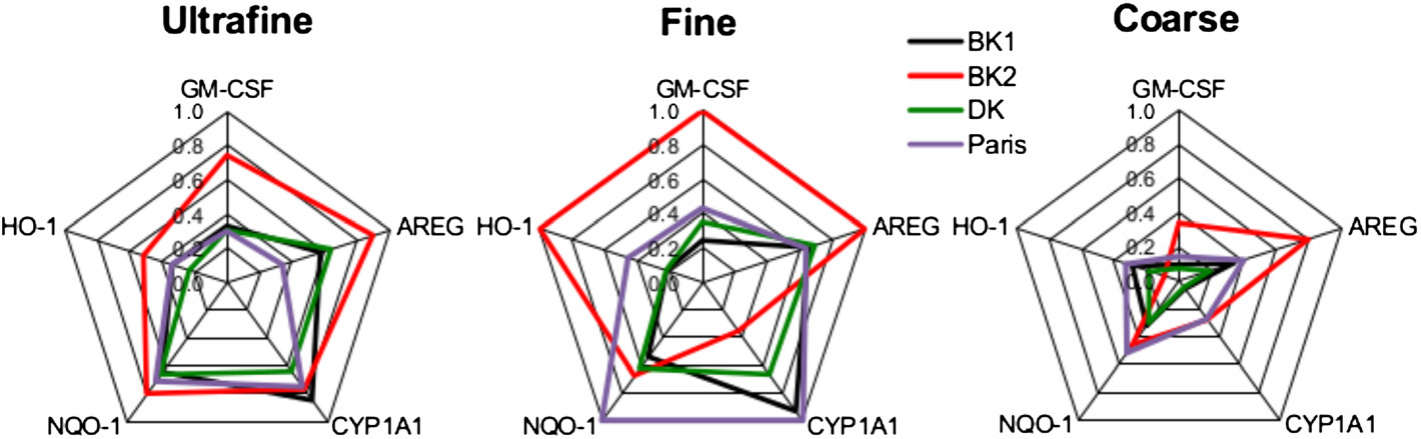
**Synthesis of African and French PM effects on 16HBE cells.** Data are represented in radar graphs in order to compare the effect of size fractions and the origin of the particles on mRNA expression. For each biomarker and whatever PM size and origin, the most induced condition is fixed to 1, and the other results are related to this value.

The effects of the 3 samples according to their size were compared with aerosols from traffic in Paris that were tested in the same conditions [16] (Figure 7). Globally, it appears that whatever the size, higher effects were observed with BK2 whereas BK1, DK and Paris exhibited lower and similar effects. For BK2, UF PM had a lower effect than F PM whereas for the other sites both size fractions had the same effect. In all the cases, C PM had a lower effect on an equal mass basis strengthening our previous observations on occidental urban and rural aerosols [15,16].

Our experiments were performed exposing cells at isomass that does not take into account the ambient level of PM. The French urban site used for comparison had a concentration of 25 μg.m^-3^, lower compared to African sites (BK1, BK2 and DK showed 206, 122 and 81 μg.m^-3 ^respectively). It means that a longer exposure is necessary in Paris to be exposed at the same concentration in African cities that reinforces the strong toxicity of BK aerosol and to a lower extent of DK aerosol.

## Conclusions

This study shows that the finest PM (< 1 μm) of African urban aerosols from three different conditions triggered adaptive responses of the airway epithelium whereas coarse PM had no or low effect. Discrimination among the different sites was highlighted with GM-CSF, a relevant pro-inflammatory cytokine for airway diseases. Bamako aerosol was characterized by an impressive biological reactivity associated with local sources as it was less reactive when diluted by external input such as dusts. PM-induced responses are related to carbonaceous aerosol content underlying the contribution of combustion sources. Regarding the prevailing sources in each site, aerosol biological impacts are higher for incomplete combustion sources from two-wheel vehicles and domestic fires, with higher relative OC content exhibiting hydrosolubility properties, than from diesel vehicles (Dakar site) with higher relative BC content. This underlines the importance of emission mitigation (e.g. composition of the traffic fleet) and the imperative need to evaluate and regulate particulate pollution in Africa. Taking into consideration PM mass quantities in the air of BK and DK sites, the African population is highly exposed to toxic particulate pollution that could lead to strong adverse health effects especially in susceptible people such as children.

## Methods

### Sampling locations

Aerosol samples were collected in Africa during the frame of the POLCA campaigns at two sampling sites: Bamako (12°39′N, 8°04′W), located in a basin in southwestern Mali, is a dusty city with 2.2 million inhabitants (2009), and Dakar (14°40′N, 17°25′W), is a coastal city in west Senegal with a population of about 3 million people (25% of the national population). Period of sampling was January 20 to 22, 2009 and January 27 to 29, 2009 in Bamako for BK1 and BK2 samples respectively and December 5 to 7, 2009 in Dakar (called DK sample). Particles were collected downtown near intense traffic roads on a 3 meter high balcony.

### PM sample collection

Aerosol collection was performed by three cascade impactors (two 13-stage electrical low pressure impactors Dekati/ELPI working at a flow rate of 30 L.min^-1^ and one of 5-stage Sioutas impactor at a flow rate of 9 L.min^-1^), running in parallel for 48 h. One of them, mounted with 25 mm diameter polycarbonate Nuclepore filters (1 μm porosity), was devoted to gravimetric measurements including number and mass size distribution, and for biological analyzes. The other impactor, mounted with 25 mm diameter quartz filters (QMA, Whatman), was dedicated to carbonaceous aerosol measurements (black carbon, BC and organic carbon, OC). An additional Sioutas impactor was mounted with 25 mm diameter Teflon filter (Zefluor, Pall Corporation) at the four first stages and 37 mm diameter at the last stage, for gravimetric measurements (mass) and water soluble ion and trace elements analysis. Aerodynamic particle diameters given by Dekati ELPI are ranged between 0.03 and 10 μm while Sioutas cascade impactor collects particles in the following size range: >0.25 μm, 1–2.5 μm, 0.5-1 μm, 0.25-0.50 μm and <0.25 μm.

**Figure 8:**
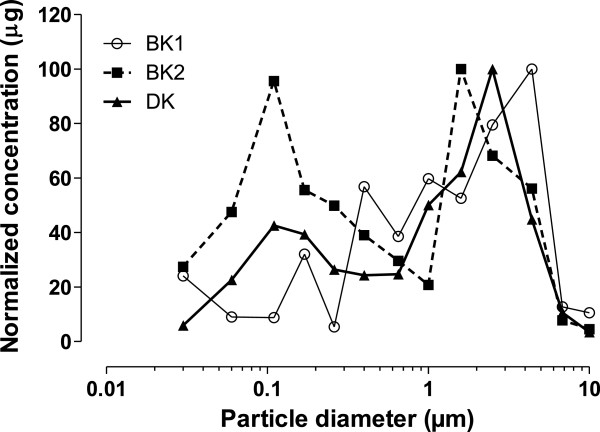
**Aerosol size distribution in Bamako (BK1 and BK2) and Dakar (DK).** PM mass was determined for each membrane of impactor. Normalized concentration (μg) is represented as a function of particle aerodynamic diameter (μm).

In accordance with mass distribution of aerosols obtained for BK and DK samples (Figure 8), three particle size fractions were selected: ultrafine particle, UF [0.03-0.1 μm], fine, F [0.1-1 μm], and coarse, C [1–10 μm].

### Chemical analyses

All Nuclepore and Teflon filters were weighed before and after sampling for mass determination, using a Mettler Microbalance MC21S with 1 μg sensitivity. One half sections of the filters were analyzed for major ion contents (Na^+^, NH_4_^+^, K^+^, Mg^2+^, Ca^2+^, SO_4_^2-^, NO_3_^-^, Cl^-^), using ion chromatographic (IC) analyser. These measurements were conducted following the analytical protocol described in Adon et al. [45]. Inductively coupled plasma mass spectrometry (ICP-MS) was applied after microwave digestion in acids (HNO_3 _and HF), to the others half sections of the filters, to determine the concentration of trace elements [46]. Black carbon (BC) and total carbon (TC) concentrations were measured on quartz filter with a thermal method developed by Cachier [20]. Prior to analyses, carbonates are removed under HCl fumes (due to carbonates interference with carbon measurements). Two similar aliquots of the same filter were then separately analyzed. One portion was directly analyzed for its total carbon content (TC). The other portion was firstly submitted to a pre-combustion step (2 h at 340°C under pure oxygen) in order to eliminate OC, and then analyzed for its BC content. Organic carbon (OC) concentrations were calculated as the difference between TC and BC.

### Aerosol chemical mass closure

PM mass closure was performed following Guinot [47] methodology in order to obtain estimates POM (Particulate Organic Matter), dust, and no determined (n.d.) mass, which is refer to the difference between the aerosols weighed mass and the reconstructed mass. In first instance, OC-to-POM conversion factor was arbitrary fixed to be 1.8 and then linear regression was performed between Ca^2+ ^concentration (obtained by IC) and the missing mass, which is calculated by the difference between the reconstructed coarse mass (sum of BC, POM and ions mass concentrations) and the weighed coarse mass. The slope of this linear regression represents Ca^2+^-to-dust conversion factor. However, OC-to-POM conversion factor is modulated in order to obtain a reconstructed mass as close as possible below the weighed mass [47,48]. OC-to-POM conversion factor is generally taken in the range 1.2 – 1.6 for urban aerosols [49,50] with higher values (1.6–2.1) for non urban aerosols [51]. In our case, OC/POM ratios are of the order of 1.63, 1.46 and 1.37 for BK1, BK2 and DK respectively.

Dust concentrations obtained by Guinot [52] methodology are confirmed with dust estimates obtained from trace element measurements [53] and calculation using the following equation:

$$\begin{array}{ll} \text{Dust} & =1.89*\mathrm{Al}+1.21*K+1.95*\mathrm{Ca}+1.66*\mathrm{Mg}\\ & \;+1.7*\mathrm{Ti}+2.14*\mathrm{Si}+1.42*\mathrm{Fe} \end{array}$$

Using these two methodologies, Guinot [54] measured dust concentrations are 88.15 (64.18), 32.48 (24.64) and 15.09 (11.04) μg.m^-3 ^in BK1, BK2 and DK, respectively.

This aerosol chemical closure was performed for each ultrafine, fine and coarse fraction of BK1, BK2 and DK samples.

### Other measurements

In parallel, aerosol particles were collected on A4 filter with high volume impactor and analyzed for 18 polar and no polar PAHs by external calibration performed with a gas chromatography coupled to a mass spectrometry following the method described in Besombes [53]. Another 5 stages high volume cascade impactor (Staplex® Model 235) was dedicated to WSOC measurements. WSOC were quantified using the protocol described by Favez [55]. Note that WSOC was also determined on Teflon filters of a Dekati impactor sampled in the same conditions (common source characteristics) but for different days than for BK2 and DK. Finally, BC concentrations measurements were also performed for UV and IR wavelengths with aethalometers in Bamako and Dakar for all the sampling periods, UV BC/IR BC ratio indicating relative “brown” like carbon or “light absorbing organic matter” fraction [56].

### Reconstitution of particle suspensions for biological experiments

For toxicological studies, the three size-fractions presented before were reconstituted from Nuclepore membranes. Recovery of the particles was achieved as previously described by Ramgolam [15]. Briefly, polycarbonate membranes were sonicated (Ultrasonic Processor, Bioblock scientific) 3 × 5 sec at 60 Watt in presence of DMEM/F12 medium (Invitrogen®) supplemented with glutamax (1%), penicillin (100 U/ml), streptomycin (100 μg/ml) and fungizone (0.125 μg/ml). Particle suspensions were stored at −20°C until use and were again sonicated (3 × 10 sec) just before dilution in the culture medium for cell exposure.

### Cell cultures

The human bronchial epithelial cell line 16HBE 14o- was kindly given by Dr D.C. Gruenert (San Francisco, California, USA). Cells were grown in DMEM/F12 culture medium (Invitrogen®) supplemented with Glutamax (1%) (Gibco) and UltroserG (2%) (Life Technologies). Cells were seeded on 75 cm^2 ^flasks or 12-wells plates (Costar®) coated by a solution containing collagen (Bovine collagen I, Vitrogen 2.9 mg.ml^-1^, BD Laboratories; human fibronectin, 1 mg/ml, BD Laboratories; Bovine Serum Albumin 1 mg.ml^-1^, Biosource; LHC medium, Biosource).

Cells were maintained in 95% humidified air with 5% CO_2 _at 37°C until 70 to 80% confluency and deprived of ultroser G or growth factors 4 h before exposure to different PM-size fractions for 24 h. Particles were used from 1 μg.cm^-2 ^to 10 μg.cm^-2^ that are non cytotoxic concentrations (data not shown), diluted in DMEM/F12 without growth factors and containing penicillin (100 μg.ml^-1^), streptomycin (100 μg.ml^-1^) and fungizone (1 μg.ml^-1^) from Sigma-Aldrich.

4 h before treatment, cells were deprived from serum and then treated with particles at 1, 5 and 10 μg.cm^-2^ for 24 h (corresponding respectively to 5.4, 27.1 and 54.3 μg.ml^-1^).

### Real-time quantitative polymerase chain reaction (qPCR)

Polymerase chain reaction (PCR) was performed to evaluate GM-CSF, IL-6, CYP1A1, NQO-1, HO-1 and AREG mRNA expression. The ribosomal protein (RPL19) gene was used as an internal control. After 24 h of treatment mRNA extraction and purification were performed using a commercially available kit (SV Total RNA Isolation System, Promega) according to the manufacturer’s recommendations. Reverse transcription was done by M-MLV (Moloney Murine Leukemia Virus) Reverse Transcriptase kit (Promega). Finally, Real-time quantitative PCR (qPCR) analysis was performed using LightCycler® 480 (Roche).

The following primer sequences were used:

RPL19: Sense: 5^′^-GGCTCGCCTCTAGTGTCCTC-3^′^

Antisense: 5^′^-CAAGGTGTTTTTCCGGCATC-3^′^

GM-CSF: Sense: 5^′^-AGCCGACCTGCCTACAGAC-3^′^

IL-6: Sense: 5^′^-ACAGCCACTCACCTCTTCAG-3^′^

Antisense: 5^′^-TGGAAGCATCCATCTTTTTC-3^′^

CYP1A1: Sense: 5^′^-GAGCCTCATGTATTTGGTGATG-3^′^

Antisense: 5^′^-TTGTGTCTCTTGTTGTGCTGTG-3^′^

NQO-1: Sense: 5^′^-AAGAAAGGATGGGAGGTGGT-3^′^

Antisense: 5^′^-GCTTCTTTTGTTCAGCCACA-3^′^

HO-1: Sense: 5^′^-CAGGCAGAGAATGCTGAGTTC-3^′^

Antisense: 5^′^-GCTCTTCTGGGAAGTAGACAGG-3^′^

AREG: Sense: 5^′^-TGGTGCTGTCGCTCTTGATA-3^′^

Antisense: 5^′^-CCCTGAAGACATCTCACTTC-3^′^

The relative quantification of the gene of interest was done according to the method described by Pfaffl [57].

### Cytokine release assay: Enzyme linked immunosorbent assay (ELISA)

After 24 h of PM treatment, the culture medium was removed, centrifuged at 10,000 *g* for 10 min at 4°C to eliminate particles, and stored at −20°C until use. GM-CSF, IL-6 and amphiregulin (AREG) amounts in supernatants were evaluated using an ELISA kit provided by R&D Systems. The optic density was measured at 450 nm with a microplate photometer MR5000 (Dynex technologies).

### Statistical analysis

Data represented as mean ± SD were evaluated by analysis of variance (ANOVA) followed by Dunnet’s t-test to examine the differences between the different treated groups with respect to the control. Correlations were calculated using Pearson’s correlation. Dendrograms were calculated using the Spearman’s dissimilarity (XLstat software).

## Abbreviations

16HBE: 16 human bronchial epithelial; ARE: Antioxidant responsive element; AREG: Amphiregulin; BaP: Benzoapyrene; BC: Black carbon; BbkF: Benzo[k]fluoranthene; BghiP: Benzo[ghi]perylene; BK: Bamako; C: Coarse; CHR: Chrysene; COPD: Chronic obstructive pulmonary disease; CYP1A1: Cytochrome P450 1A1; DK: Dakar; ELISA: Enzyme linked immunosorbent assay; F: Fine; FLUA: Fluoranthene; GM-CSF: Granulocyte macrophage-colony stimulating factor; HO-1: Heme oxygenase 1; IARC: International agency for research on cancer; IL-6: Interleukin 6; IncdP: Indeno[1,2,3-cd]pyrene; UV: Ultraviolet; IR: Infrared; NHBE: Normal human bronchial epithelial; NQO-1: NADPH quinine oxydoreductase 1; OC: Organic carbon; PCR: Polymerase chain reaction; PHE: Phenanthrene; PM: Particulate matter; POLCA: Pollution des capitales africaines; POM: Particulate organic matter; PYR: Pyrene; UF: Ultrafine; RPL-19: Ribosomal protein L19; TC: Total carbon; WSOC: Water soluble organic carbon; XME: Xenobiotic responsive element.

## Competing interests

The authors declare no conflict of interest.

## Authors’ contribution

SV performed the biological analyses, interpreted the results and contributed to write the paper. CL designed the research and with EHTD performed field experiments, analyzed the data and drafted the paper. CGL designed the research and performed field experiments with EG. HC contributed to the interpretation of the data. EHTD, HC, NM, EGand AS performed the chemical analysis. AB provided advices for statistical analysis. ABS directed the joint project and contributed to write the paper. All authors read and approved the manuscript.

## Supplementary Material

Additional file 1 **Correlation coefficients (Pearson) between biomarker responses induced by Parisian PM exposure.** Correlations were calculated with results of Val et al. [16] for UF and F fractions as well as all size fractions (Paris). Bold values represent a statistical correlation between biomarkers (p<0.05). (PDF 209 kb) Click here for file
