# Supplementary material for: Physico-chemical characterization of African urban aerosols (Bamako in Mali and Dakar in Senegal) and their toxic effects in human bronchial epithelial cells: description of a worrying situation
Source: Part Fibre Toxicol. 2013 Apr 2;10:10. doi: 10.1186/1743-8977-10-10 (PMC3637552; doi:10.1186/1743-8977-10-10)
Supplement: Additional file 1 — Correlation coefficients (Pearson) between biomarker responses induced by Parisian PM exposure. Correlations were calculated with results of Val et al. [16] for UF and F fractions as well as all size fractions (Paris). Bold values represent a statistical correlation between biomarkers (p<0.05). (PDF 209 kb) [file 1743-8977-10-10-S1.pdf]

Additional file 1: **Correlation coefficients (Pearson) between biomarker responses induced by Parisian PM exposure.** Correlations were calculated with results of Val et al. [16] for UF and F fractions as well as all size fractions (Paris). Bold values represent a statistical correlation between biomarkers ($p<0.05$).

**Paris UF**	GM-CSF	IL-6	AREG	CYP1A1	NQO-1	HO-1
GM-CSF	**1**					
IL-6	**0.831**	**1**				
AREG	0.463	0.497	**1**			
CYP1A1	**0.767**	0.553	0.326	**1**		
NQO-1	**0.770**	0.546	0.374	**0.895**	**1**	
HO-1	**0.893**	**0.749**	0.364	**0.823**	**0.785**	**1**

**Paris F**	GM-CSF	IL-6	AREG	CYP1A1	NQO-1	HO-1
GM-CSF	**1**					
IL-6	**0.893**	**1**				
AREG	**0.894**	**0.819**	**1**			
CYP1A1	**0.804**	**0.754**	**0.843**	**1**		
NQO-1	**0.678**	**0.612**	**0.765**	**0.907**	**1**	
HO-1	**0.789**	**0.621**	**0.787**	**0.882**	**0.934**	**1**

**Paris**	GM-CSF	IL-6	AREG	CYP1A1	NQO-1	HO-1
GM-CSF	**1**					
IL-6	**0.848**	**1**				
AREG	**0.788**	**0.710**	**1**			
CYP1A1	**0.830**	**0.709**	**0.722**	**1**		
NQO-1	**0.753**	**0.647**	**0.727**	**0.884**	**1**	
HO-1	**0.701**	**0.633**	**0.671**	**0.756**	**0.804**	**1**
